# ActIIR inhibition improves motor outcome and preserves muscle fibers after experimental autoimmune neuritis

**DOI:** 10.1186/s40478-026-02277-z

**Published:** 2026-03-27

**Authors:** Ivo Gerlach, Manuel Koch, Graziana Gatto, Jan-Wilm Lackmann, Helmar C. Lehmann, Michael Schroeter, Felix Kohle

**Affiliations:** 1 Department of Neurology, Faculty of Medicine, University of Cologne and University Hospital Cologne, Kerpener Strasse 62, 50937 Cologne, Germany; 2 Center for Biochemistry, Institute for Dental Research and Oral Musculoskeletal Research, Faculty of Medicine and University Hospital Cologne, University of Cologne, Cologne, Germany; 3 Faculty of Mathematics and Natural Sciences, CECAD Cluster of Excellence, University of Cologne, Cologne, Germany; 4 Department of Neurology, Hospital Leverkusen, Leverkusen, Germany

**Keywords:** Experimental autoimmune neuritis, Guillain-Barré syndrome, Muscle atrophy, Activin receptors, Ubiquitin–proteasome system, Ubiquitin-protein ligases, Recovery of function, Denervation

## Abstract

Secondary muscle atrophy resulting from neuronal denervation is a key determinant of poor functional recovery in immune-mediated neuropathies, even after inflammation is resolved. While current immunotherapies effectively limit immune-mediated nerve injury, secondary muscle atrophy as a downstream consequence of denervation is not addressed by existing treatment strategies. Activin type II receptors (ActIIR) are central regulators of skeletal muscle atrophy, and pharmacological inhibition of ActIIR signaling has shown variable efficacy in primary myopathies. Here, we investigated the therapeutic potential of ActIIR inhibition in promoting motor recovery in immune-mediated neuropathies using experimental autoimmune neuritis, a rodent model of Guillain-Barré syndrome. At the onset of the recovery phase, animals received varying doses of a synthesized anti-ActIIR antibody. Motor performance was assessed using a standardized clinical neuritis score, grip strength testing, electrophysiological nerve conduction studies, and balance-beam performance combined with quantitative kinematic gait analysis. High-dose ActIIR antibody treatment significantly improved motor performance during the recovery phase. This improvement was not associated with detectable changes in peripheral nerve immune infiltration, inflammatory cytokine expression, or myelination, but correlated with preservation of muscle fiber size. Consistent with this, muscle proteomic and targeted transcriptional analyses revealed attenuation of FoxO-dependent atrophy programs and reduced expression of the E3 ubiquitin ligases Atrogin-1 and MuRF1. Together, these findings suggest that ActIIR inhibition limits secondary muscle atrophy and enhances motor recovery in autoimmune neuritis. Targeting ActIIR signaling may therefore represent a promising muscle-directed adjunct strategy to improve motor outcomes in immune-mediated neuropathies.

**Supplementary Information:**

The online version contains supplementary material available at 10.1186/s40478-026-02277-z.

## Introduction

Skeletal muscles are essential not only for motor execution but also as major metabolic reservoirs, e.g., for protein, glucose, and lipid homeostasis. Muscle cells are highly adaptive, altering myofiber size in response to metabolic or mechanical cues. While anabolic stimuli, such as exercise and androgens, induce hypertrophy, catabolic states and degenerative diseases result in atrophy. Muscle atrophy is classified as primary, when degeneration originates within the muscle itself, or secondary, when caused by extrinsic factors, e.g. disuse or denervation [82]. Neurogenic, denervation-induced atrophy is a common feature of many neurological disorders, including traumatic nerve injuries and peripheral neuropathies (e.g., Guillain-Barré syndrome (GBS)) [23].

The extent and duration of muscle denervation define the degree of recovery, with prolonged denervation resulting in irreversible muscle fiber loss [9, 37]. This principle also extends to immune-mediated neuropathies, where secondary neurogenic atrophy following inflammation-induced nerve damage determines long-term motor recovery [74]. Although the peripheral nervous system exhibits considerable neuroregenerative capacity, motor recovery in GBS and chronic immune-mediated neuropathies, e.g. chronic inflammatory demyelinating polyneuropathy (CIDP), is frequently incomplete, significantly contributing to disease burden [29, 72, 74]. Early initiation of immunotherapy is essential in both GBS and CIDP, as it reduces denervation, allowing timely reinnervation, and shortening the window during which irreversible muscle loss can occur [62, 66, 70, 73, 94].

Strategies to preserve muscle integrity during this critical period of reinnervation may therefore improve functional recovery. However, while most research in immune neuropathies focuses on diagnostic optimization and immunopathogenesis, the prevention of secondary muscle atrophy has received comparatively little attention. By contrast, therapeutic modulation of skeletal muscle atrophy has been extensively studied in primary myopathies and degenerative conditions such as spinal muscular atrophy [44]. These studies have identified the myostatin pathway, acting through Activin type II receptors (ActIIR), as a key regulator of muscle growth and atrophy [47, 59, 60]. For example, Bimagrumab, a monoclonal antibody that blocks ligand binding to both ActIIR A and B, inhibits myostatin, activin A/B, and GDF-11 signaling, thereby inhibiting signaling pathways implicated in muscle atrophy [64]. Preclinical and clinical studies have demonstrated that pharmacological ActIIR inhibition increases muscle mass following immobilization and improves metabolic parameters in patients with type 2 diabetes, with a favorable safety profile [32, 43, 64, 76]. However, whether blocking ActIIRs might also be beneficial in limiting secondary, immune nerve injury-mediated muscle atrophy remains unclear.

Here, we report that ActIIR inhibition preserves muscle fiber size, is associated with reduced expression of the atrophy-related genes MuRF1 and Atrogin-1, and significantly improves motor recovery in the experimental autoimmune neuritis (EAN) model of GBS [85]. By targeting a downstream effect of immune-mediated nerve injury, these findings suggest that secondary muscle atrophy limits functional recovery and that ActIIR signaling represents a muscle-directed therapeutic target to enhance motor outcome in immune-mediated neuropathies.

## Material and Methods

### EAN induction

Overall, 31 female Lewis rats (6–8 weeks old; 160–200 g) were purchased from Charles River (Sulzfeld, Germany) and housed under standardized pathogen-free conditions with ad libitum access to food and water (12 h light/dark cycle). The study consisted of an initial dose-finding experiment followed by independent replication experiments. Animals were randomly assigned to treatment groups on the day of EAN induction. All personnel involved in animal care and outcome assessment were blinded to treatment allocation.

In the dose-finding experiment, rats were allocated to three groups (ActIIR-antibody (AB) 5 mg/kg, ActIIR-AB 1.25 mg/kg, and isotype control HIV1-AB 5 mg/kg; n = 4 per group). Based on the dose-finding results, all subsequent experiments compared ActIIR-AB (5 mg/kg) versus sham (HIV-AB (5 mg/kg)). For the main outcomes (clinical neuritis score and electrophysiology), data were pooled across independent experiments to yield a total of n = 12 rats per group. Grip strength and gait analysis were performed after the dose-finding experiment (n = 8 per group). Proteomics was performed on tibialis anterior muscles from n = 4 rats per group at day 30 post immunization (30 dpi) and from an additional n = 3 EAN rats at 18 dpi. For disease induction, the rats received a subcutaneous injection of 250 µg of the neuritogenic P2^53-78^ peptide (#AS-65472, Anaspect, California, USA) dissolved in the same concentration of complete Freund's adjuvant (Sigma Aldrich, Missouri, USA) above the root of the tail. Animals were anesthetized intraperitoneally with xylazine (Inresa, Freiburg, Germany) and ketamine (Bayer, Leverkusen, Germany) (10 mg/kg and 50 mg/kg, respectively). The caretakers were blinded to treatment groups and the rats were treated with tramadol (Gruenenthal, Aachen, Germany) 0.5 mg/ml per os from the immunization day onwards to relieve potential disease burden from painful autoimmune neuritis. Animals were weighed and assessed for disease severity daily by a blinded investigator (FK) using an established neuritis score [24, 35, 70]: 0 normal; 1 less lively; 2 impaired righting/limb tail; 3 absent righting; 4 ataxic gait, abnormal position; 5 mild paraparesis; 6 moderate paraparesis; 7 severe paraplegia; 8 tetraparesis; 9 moribund; 10 death. Termination criterion was a score ≥ 7 according to animal welfare guidelines.

### Sex as a biological variable

Our study exclusively examined female rats, as female Lewis rats ensure a more robust and reproducible disease model [58].

### AB-treatment

The animals were treated subcutaneously with 5 mg/kg and 1.25 mg/kg of an ActIIR-AB and an HIV1-AB with no known reactivity to rat antigen (5 mg/kg) as a control dissolved in 1 × PBS. As we wanted to explore potential effects on the recovery phase of EAN, the animals were treated at 18 dpi at the peak of the disease course, simultaneously marking the onset of the recovery period. Treatment was repeated at 22 and 26 dpi.

### AB production

The Sleeping Beauty transposon system was applied to produce the two antibodies (light and heavy chain) for the in vivo experiments. Constructs included: heavy chain anti ACTR2B (Sequence 146 from patent US 8388968; AGM83815; AA 1–445; A232L; A233L; D354E; L356M; BM40 signal peptide; C-terminal Twin-Strep-tag), light chain anti ACTR2B (Sequence 141 from patent US 8388968; AGM83810; AA 1-106; BM40 signal peptide and constant part S52059; AA: 25-135), heavy chain anti HIV (Sequence 1 from patent US 11845787; WVT46656; AA 20–472; D381E; L383M; signal peptide AAA86305: AA 1-19; C-terminal Twin-Strep-tag), and light chain anti HIV (Sequence 13 from patent US 11845787; WVT46662; AA 21-226; N189S; signal peptide AAA86305: AA 1-21). The ACTR2B antibody sequences correspond to the bimagrumab antibody described in patent US 10982000 and have been reported to mediate dual inhibition of ActIIRA (ACVR2A) and ActIIRB (ACVR2B). Thereby, the ligand-receptor interaction of Myostatin and Activin A was shown to be disrupted [43, 64]. Given that these ligands bind to the shared ActIIRA/B ligand-binding interface, receptor blockade is therefore mechanistically expected to attenuate signaling of additional high-affinity ActIIR ligands, such as GDF11 and activin B, although this was not directly tested in the present study [78, 90]. Recombinant antibody production was performed by co-transfecting the light and heavy chain expression vectors and the transposase plasmid at a 10:1 ratio into HEK293 EBNA cells using FuGENE® HD transfection reagent (Promega GmbH, Wisconsin, USA). Post-transfection, cells underwent puromycin selection (2 µg/mL; Sigma-Aldrich, Missouri, USA) for four days and were then expanded into triple flasks. The antibody expression was induced in confluent triple flasks with doxycycline (0.5 µg/mL; Sigma-Aldrich, Missouri, USA). Supernatants from confluent cultures were collected every two days, filtered, and purified using Strep-Tactin® XT resin (IBA Lifescience, Göttingen, Germany). Purified recombinant antibodies were eluted using biotin-containing buffer (IBA Lifescience, Göttingen, Germany), dialyzed against PBS, and stored at − 80 °C.

### Nerve conduction studies

Motor nerve conduction studies were performed before the immunization at 0, 18 and 30 dpi to determine compound muscle action potential (CMAP) and motor nerve conduction velocity (MNCV) as described in [38, 40].

### Grip strength

We assessed the grip strength of all rats at 0, 18 and 30 dpi. Each rat was allowed to grab on a grid with all four limbs. The grid was attached to a force transducer that records the peak force while the rat is pulled by the tail horizontally away from the grid (BIO-GS3 Grip Test, Vitrolles, France). Three consecutive pulls separated by 20 s pauses between each pull were done. The pulls were averaged to calculate the absolute grip strength (in Newton) which we divided by the rat body weight in grams.

### Gait analysis

The rats walked on a narrow 30 mm wide and 100 cm long and flat beam. This task [42] requires some postural control but is readily doable in healthy conditions [4]. Each walk was captured with an iPhone 14 camera placed perpendicularly to the beam at 0, 18, and 30 dpi (24 frames/sec). Rats were trained to cross the beam thrice before day 0. At 0, 18 and 30 dpi each rat crossed the beam twice, therefore markerless coordination tracking in both directions was captured, so our data represent averages of both right and left limbs. We used DeepLabCut [56] to track the iliac crest, hip, knee, ankle, hindpaw, shoulder, elbow, wrist, forepaw, tail tip/center and base coordinates, as well as the beam surface to define our vertical baseline. To train the DeepLabCut model [56], we used ~ 400 image frames from different time points. To predict hindlimb kinematic positions, we manually labeled different body parts in all ~ 400 image frames. The model was trained using a deep residual network structure (ResNet-50), based on the pretrained model weights from DeepLabCut. For refinement, the outlier extraction function was used and machine-learning labeling manually refined and the network retrained until machine-labeling achieved satisfactory prediction of joint kinematics. Begin and end time points for swing and stance were identified manually for each step as well as slips. Overall, 8 rats each for the 5 mg/kg treatment group and for the control group were tracked. Step cycles were further analyzed using AutoGaitA [33]. For analysis of y-coordinates at 18 dpi, hindlimb joints were normalized to iliac crest height at 0 dpi at the end of the stance phase. Slips or pauses on the beam were excluded from further kinematic analyses.

### Immunohistochemistry

After transcardial perfusion with 1 × PBS at 30 dpi, both sciatic nerves, as well as the tibialis anterior muscles were dissected. The right sciatic nerves were cut into four segments and sorted from proximal to the distal part and the segments were immediately embedded in Tissue-Tek OCT Compound (Sakura, Tokyo, Japan) and stored in a − 80 °C freezer. The nerves and mid belly sections of the tibialis anterior muscles were cut into 12 µm thick slices on a cryostat (Leica Biosystems, Wetzlar, Germany) and 8 slices each were mounted on slides. For immunohistochemical staining, cryostat sections were thawed for 2 h at 20 °C and then fixed in acetone for 20 min at 20 °C. They were washed with 1 × PBS for five minutes. For assessment of the inflammation of the sciatic nerves the following antibodies were used:

anti-Iba1 (1:500, rabbit polyclonal, FUJIFILM Wako Shibayagi Cat# 019-19741, RRID:AB_839504, Osaka, Japan).

anti-CD3 (1:500, rabbit monoclonal antibody, Abcam Cat# ab16669, RRID:AB_443425, Cambridge, UK).

For immunohistochemical quantification of peripheral nerve myelination, FluoroMyelin Red fluorescent stain (1:300, Thermo Fisher Scientific Cat# F34652, RRID:AB_2572213, Massachusetts, USA) and anti-S100 (1:400, rabbit polyclonal, Sigma-Aldrich Cat# S2644, RRID:AB_477501, Missouri, USA) in combination with anti-Neurofilament 160/200 antibody (1:400, mouse monoclonal, Sigma-Aldrich Cat# N2912, RRID:AB_477262, Missouri, USA) were used.

To assess muscle morphology, the following antibodies were used:

anti-Slow Skeletal Myosin Heavy chain (1:500, mouse monoclonal, Abcam Cat# ab11083, RRID:AB_297734, Cambridge, UK).

anti-Fast Myosin Skeletal Heavy chain (1:500, rabbit polyclonal, Abcam Cat# ab91506, RRID:AB_10714690, Cambridge, UK).

anti-Laminin (1:400, rabbit polyclonal, Invitrogen Cat #PA1-16730, RRID:AB_2133633, Massachusetts, USA).

anti-PAX7 (1:300, mouse monoclonal, Thermo Fisher Scientific, Cat #5081-MSM1-P1ABX, RRID:AB_3697700, Massachusetts, USA).

Corresponding fluorescein-labelled secondary antibodies were applied:

goat anti-rabbit immunoglobulin G (IgG) (Alexa Fluor 488, Thermo Fisher Scientific Cat# A32731, RRID:AB_2633280, Massachusetts, USA).

goat anti-mouse IgG (Alexa Fluor 488, Jackson ImmunoResearch Labs Cat# 115–545-003, RRID:AB_2338840 Cambridge, UK).

goat anti-mouse IgG (Cy3, AffiniPure, Jackson ImmunoResearch Labs Cat# 115–165-166, RRID:AB_2338692, Cambridge, UK).

goat anti-rabbit IgG (Alexa Fluor 568, Thermo Fisher Scientific Cat# A11011, RRID:AB_143157 Massachusetts, USA).

goat anti-mouse IgG (Alexa Fluor 568, Thermo Fisher Scientific Cat# A21124, RRID:AB_2535766 Massachusetts, USA).

We used Hoechst 33,342 (1:500, Cell Signaling Technology Cat# 4082, RRID:AB_10626776, Massachusetts, USA) for cell nuclei staining. Fluorescent signals were detected using an inverted fluorescence BZ-9000 microscope (Keyence, Japan) with a 20 × or 40 × magnification numerical aperture objective lens (Nikon, Japan). Ten pictures of each slide were randomly chosen. Cell counting per section was determined using image analysis software (ImageJ, Fiji, open source program) with a semi-quantitative approach [16] for the cellular inflammation markers and muscle morphology was assessed via cross-sectional area (CSA), minimal Feret’s diameter via ImageJ and the plugin MuscleJ [8, 57]. Only fibers with intact laminin borders were included in the analysis. Central nuclei and Pax7-positive cells were counted manually. All slides were mounted using Fluoromount G mounting-medium (Biozol, Eching, Germany).

### Proteomics analysis

After perfusion at 30 dpi, n = 4 left tibialis anterior muscles of the sham group and the 5 mg/kg ActIIR-AB-treatment group were extracted and dissected into smaller pieces. In three additional female Lewis rats, EAN was induced and they were perfused at 18 dpi to extract the tibialis anterior muscles. 250 µL of RIPA buffer was added and the tissue homogenized via ultrasound sonication. The samples were then allocated. One sample was used for BCA protein assay (Thermo Fisher Scientific, Waltham, Massachusetts, USA) to define protein amounts in the muscle sample. Absorbance was measured in a plate reader at 560 nm and the protein concentrations of the samples were calculated by using the BSA reference absorbance.

#### Protein digestion for LC–MS/MS

The aliquots were further lysed by adding 200 µl ice-cold Urea lysis buffer (8 M Urea in 50 mM Triethylammoniumbicarbonate supplemented with 50 × protease inhibitor cocktail (Roche, Mannheim, Germany)). Supernatants were collected after repetitive vortexing and centrifugation, incubated with 25 Units Benzonase HC for 30 min at 37 °C to degrade nucleic acids and centrifuged. 50 µg of protein per sample were transferred to a 1.5 ml tube and incubated with Dithiothreitol to a final concentration of 5 mM for 1 h at room temperature. In a next step, the samples were incubated with Chloroacetamide at a final concentration of 40 mM for 30 min in the dark and, subsequently, with Lys-C protease at a ratio of 1:75 for 4 h. 50 mM TEAB was added to achieve a final concentration of Urea ≤ 2 M and the samples were incubated with Trypsin (1:75) overnight. Formic acid at a final concentration of 1% was added to stop enzymatic digestion. Peptides were purified with SDB-RPS polymer sorbent StageTips (CDS Analytical, Oxford, Pennsylvania, USA).

#### Data acquisition

Samples were analyzed by the CECAD Proteomics Facility on an Orbitrap Exploris 480 (Thermo Scientific, granted by the German Research Foundation under INST 1856/71–1 FUGG) mass spectrometer equipped with a FAIMSpro differential ion mobility device that was coupled to an UltiMate 3000 (Thermo Scientific). Samples were loaded onto a precolumn (Acclaim 5 µm PepMap 300 µ Cartridge) for 2 min at 15 μl flow before reverse-flushed onto an in-house packed analytical column (30 cm length, 75 µm inner diameter, filled with 2.7 µm Poroshell EC120 C18, Agilent). Peptides were chromatographically separated at a constant flow rate of 300 nL/min and the following gradient: initial 6% B (0.1% formic acid in 80% acetonitrile), up to 32% B in 72 min, up to 55% B within 7.0 min and up to 95% solvent B within 2.0 min, followed by column wash with 95% solvent B and reequilibration to initial condition. The FAIMS pro was operated at − 50 V compensation voltage and electrode temperatures of 99.5 °C for the inner and 85 °C for the outer electrode. MS1 scans were acquired from 399 to 1001 m/z at 15 k resolution. Maximum injection time was set to 22 ms and the AGC target to 100%. MS2 scans ranged from 400 to 1000 m/z and were acquired at 15 k resolution with a maximum injection time of 22 ms and an AGC target of 100%. DIA scans covering the precursor range from 400–1000 m/z and were acquired in 60 × 10 m/z windows with an overlap of 1 m/z. All scans were stored as centroid.

#### Sample processing in DIA-NN

Samples were analyzed in DIA-NN 1.8.1 [19]. Uniprot Swissprot and TrEMBL rat databases (UP2494, downloaded 15/01/25) were merged and used for library building with settings matching acquisition parameters and the match-between-runs function enabled [13]. Here, samples are directly used to refine the library for a second search of the sample data. DIA-NN was run with the additional command line prompts “—report-lib-info”. Further output settings were: filtered at 0.01 FDR, N-terminal methionine excision enabled, maximum number of missed cleavages set to 1, min peptide length set to 7, max peptide length set to 30, min precursor m/z set to 400, max precursor m/z set to 1000, cysteine carbamidomethylation enabled as a fixed modification. Afterwards, DIA-NN output was further filtered on library q-value and global q-value <  = 0.01 and at least one peptide per protein using R (4.1.3). Finally, LFQ values calculated using the DIA-NN R-package. Afterwards, analysis of results was performed in Perseus 1.6.15 [93]. Student’s t test was used to test for significant (*p* < 0.05) changes in protein abundance across the biological conditions. GO terms were added to the dataset. The relative enrichment of GO terms in the group of significantly changed proteins was determined using Fisher’s exact test. The whole dataset was inserted into the STRING-database to explore any protein-network interactions [88, 92] in the organism *Rattus norvegicus*.

#### Mapping of differentially expressed proteins to FOXO/SMAD chromatin immunoprecipitation-Atlas targets

Significantly changed proteins at 30 dpi (log_2_FC > 1 or < − 1, *p* < 0.05) were mapped to rat gene symbols to assess their association with transcriptional regulatory programs involving FOXO and SMAD transcription factors, using publicly available chromatin immunoprecipitation datasets from ChIP-Atlas 3.0 [100]. As muscle-specific ChIP-seq datasets for rat are limited, rat genes were mapped to mouse and human orthologs using Ensembl BioMart (Ensembl Genes, *rnorvegicus*_gene_ensembl), based on Ensembl gene identifiers [22]. Only one-to-one ortholog mappings were retained. For each species (rat rn6, mouse mm10, human hg38), ChIP-Atlas target gene tables were retrieved for FOXO1, FOXO3, FOXO4, and for the canonical SMAD transcription factors SMAD2, SMAD3, and SMAD4. ChIP-Atlas target files summarize chromatin immunoprecipitation*-*seq peak enrichment aggregated across multiple experiments and report gene-level binding scores within defined genomic windows relative to transcription start sites. To focus on promoter-proximal transcription factor binding, only target gene annotations within ± 1 kb of transcription start sites were considered. To enrich for muscle-relevant regulatory contexts, target gene annotations were further filtered based on tissue- and lineage-associated ChIP-Atlas columns using keyword matching (including myoblast, myotube, skeletal muscle, muscle lineage, C2C12, MyoD, Myogenin, and Pax7). Genes meeting both promoter proximity and tissue-filtering criteria were defined as FOXO- or SMAD-associated for a given transcription factor. For cross-species analyses, genes were required to satisfy *all* of the following criteria simultaneously in *both* mouse and human datasets: (i) promoter-proximal binding within ± 1 kb of the transcription start site, (ii) binding by at least one FOXO family member (FOXO1, FOXO3, or FOXO4), and (iii) binding by all three canonical SMAD transcription factors (SMAD2, SMAD3, and SMAD4).

### Quantitative real-time PCR

RNA of the left sciatic nerves and left tibialis anterior muscles (n = 8 per each group) were isolated by using the MasterPureTM Complete DNA & RNA Purification Kit (Lucigen, Hoddesdon, United Kingdom) and stored at − 20 °C. We used the QuantiTect Reverse Transcription Kit (Qiagen, Hilden, Germany) to synthesize the cDNA of the RNA as described [36]. The amplification reactions for MuRF1 and Atrogin-1 were carried out with a program involving a step at 95 °C for 1 min followed by 40 cycles of 95 °C for 15 s, 60 °C for 30 s and 72 °C for 30 s. The amplification reactions for all cytokines were: 95 °C for 1 min followed by 40 cycles of 95 °C for 15 s, 56 °C for 15 s and 72 °C for 45 s. The used primers are provided in the Supplemental Table 1.

### Statistical methods

Statistical analyses were performed by Prism software (GraphPad Prism 10, San Diego, California, USA). Unless stated otherwise, data are provided as mean ± SEM. The data were tested for normality to meet the assumptions of the statistical test. For normally distributed data, an unpaired t-test with Welch’s correction was used. Differences between three or more groups were tested by one-factor analysis of variance (ANOVA). For non-normally distributed data, Mann–Whitney test was used. Nested t-test was applied to immunohistology data with technical replicates. In all experiments, a *p*-value of < 0.05 was defined as statistically significant, and *p* < 0.0001 was considered highly statistically significant.

## Results

### High-dose ActIIR inhibition improves functional motor recovery in EAN

**Fig. 1:**
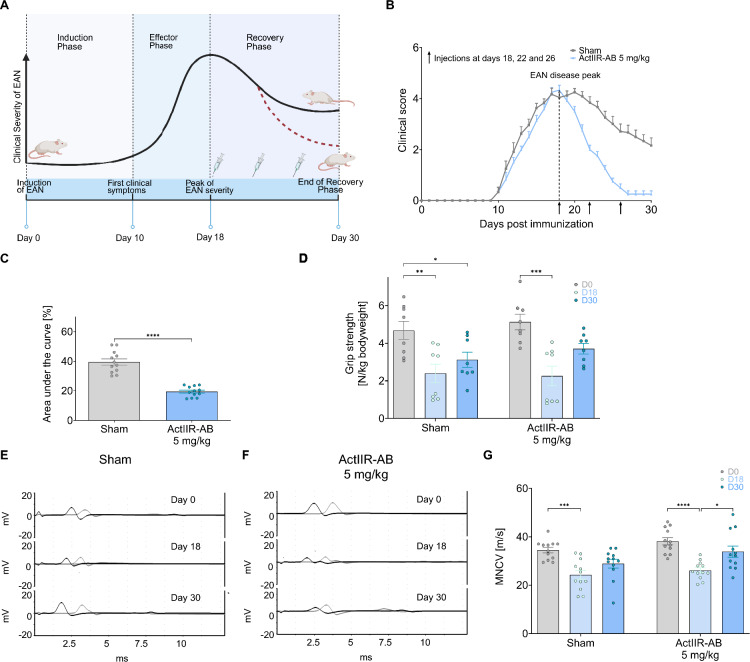
High-dose ActIIR inhibition improves motor recovery in EAN. **A** Schematic of the experimental design. Experimental autoimmune neuritis (EAN) was induced on day 0 (dpi, days post immunization). Clinical symptoms peaked around 18 dpi, marking the transition to the recovery phase. Anti-ActIIR-AB or anti-HIV-AB (sham) was administered subcutaneously on 18, 22, and 26 dpi. **B** Disease course across all experiments for sham-treated and high-dose (5 mg/kg) anti-ActIIR-AB-treated animals. **C** AUC analysis for the recovery phase (18 to 30 dpi) comparing sham and high-dose treatment groups. **D** Grip strength was assessed at baseline (0 dpi), neuritis peak (18 dpi) and the end of recovery phase (30 dpi). Motor nerve conduction studies, exemplarily shown in (**E**) for sham-treated animals and in (**F**) for the anti-ActIIR-AB-treatment group for nerve conduction velocity (MNCV) (**G**). Behavioral and electrophysiological data for the high-dose (5 mg/kg) and sham groups were replicated in three independent experiments (total n = 12 per group), including the dose titration experiment (see Supplemental Fig. 1). Grip strength was assessed in two independent experiments (n = 8 overall per group). Data are plotted as mean ± SEM. Differences between three or more groups were tested by one-factor analysis of variance (ANOVA) followed by the Holm-Sidak multiple-comparison test. An unpaired t-test with Welch’s correction was used for (**C**). *P < 0.05, **P < 0.01, ***P < 0.001, ****P < 0.0001

In our rat model of EAN, disease progression was characterized using a standardized neuritis score, which quantifies ascending motor deficits and gait ataxia [24, 35]. The score ranges from impaired righting reflex to severe paraplegia, resembling the ascending paralysis observed in patients with GBS. Following immunization (0 dpi), clinical symptoms emerged around 10 dpi and peaked by 18 dpi, marking the effector phase (Fig. 1A). At disease peak, the mean clinical score was comparable between groups, with animals showing mild to moderate hindlimb paresis and ataxia (Fig. 1B, and Supplemental Fig. 1A). During the recovery phase, there was a gradual improvement of the motor deficits (Fig. 1, A and B, and Supplemental Fig. 1A). To evaluate the rehabilitative potential of ActIIR blockade, we treated rats with an ActIIR-AB at the peak of EAN at 18 dpi, simultaneously marking the onset of the recovery phase. Based on reported ActIIR-AB dosages [3, 30, 64, 69], we conducted a dose-titration experiment comparing subcutaneous high (5 mg/kg) and low (1.25 mg/kg) doses of the ActIIR-AB (Supplemental Fig. 1A). Sham-treated rats received an anti-HIV1-AB (5 mg/kg). Only the high dose of ActIIR-AB resulted in a significantly improved motor recovery, as reflected by reduced neuritis scores (Supplemental Fig. 1, A and B) and increased weight gain (Supplemental Fig. 1C). Thus, subsequent experiments were conducted using only the high dose.

By 18 dpi rats showed the highest neuritis score, reduced grip strength, a validated metric for disease severity and functional recovery in GBS [21], and decreased MNCV (Fig. 1, D-G, and Supplemental Fig. 1D). By 30 dpi, ActIIR-AB treatment (5 mg/kg) led to a significant improvement of motor deficits as assessed by the area under the curve (AUC) defined by the neuritis score (Fig. 1, B and C), reverted grip strength to baseline levels (Fig. 1D), and partially rescued MNCV (Fig. 1, E–G). No differences in CMAP were detected (Supplemental Fig. 1D).

### Kinematic gait analysis reveals improved postural recovery

**Fig. 2:**
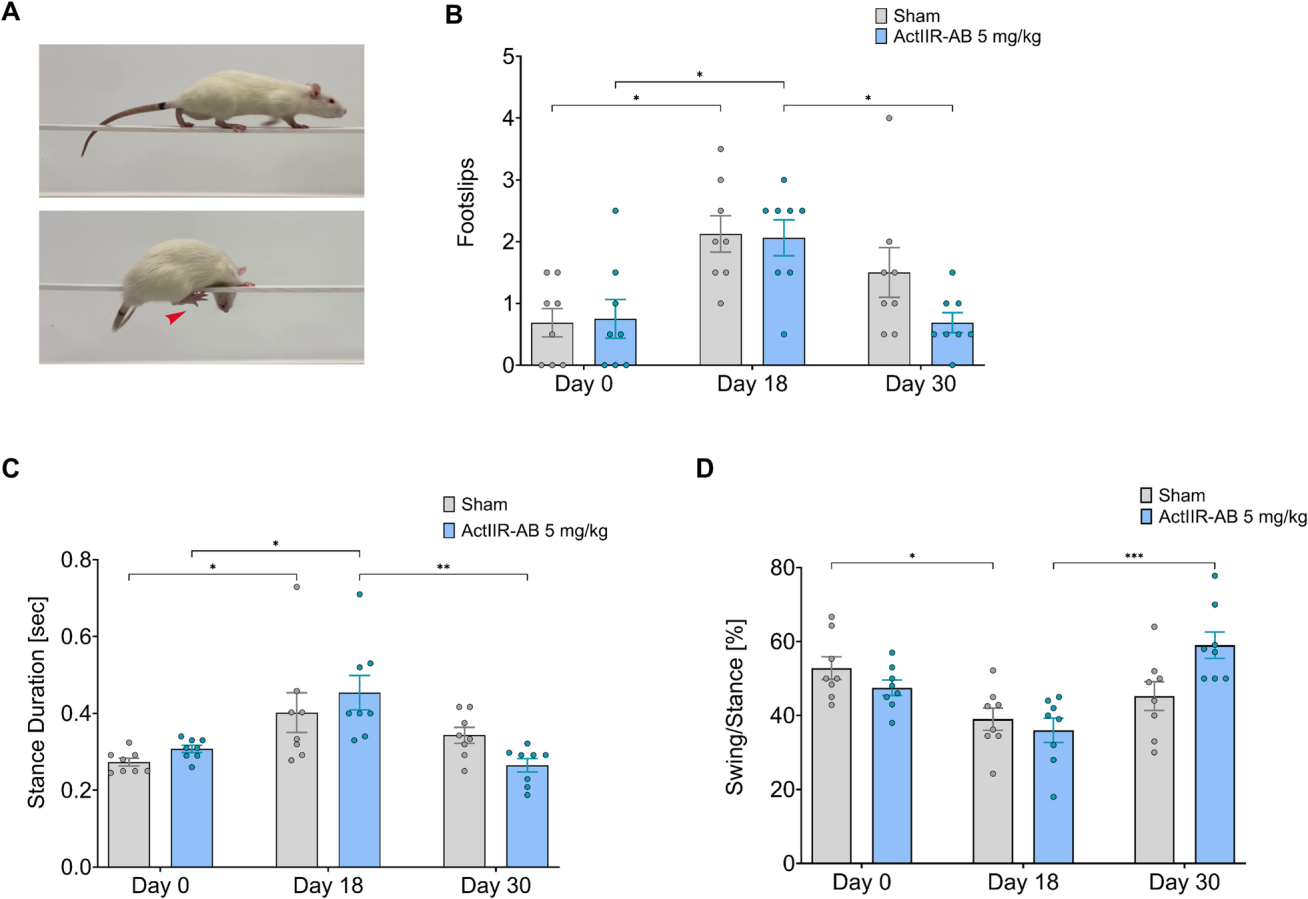
EAN is characterized by loss of postural control. **A** Video tracking of rats crossing a 30 mm beam at 30 days post immunization (30 dpi). The top image shows an anti-ActIIR-AB treated rat (5 mg/kg), and the lower image a representative footslip of a sham-treated rat. **B** Quantification of foot slips across EAN timeline: baseline (0 dpi), disease peak (18 dpi), and the end of recovery (30 dpi) in sham- and anti-ActIIR-AB treated animals. **C** Stance duration per step cycle across the disease timeline. **D** Bar graph depicting the percentage of the step cycle spent in the swing phase. Balance beam gait analysis was performed on n = 8 rats per group (EAN induced in two independent experiments with n = 4 per group each). Data are plotted as mean ± SEM. Differences between three or more groups were tested by one-factor analysis of variance (ANOVA) followed by the Holm-Sidak multiple-comparison test. *P < 0.05, **P < 0.01, ***P < 0.001

Gait ataxia and postural instability due to paresis and proprioceptive deficits are hallmarks of GBS, and are also observed in EAN [48, 75]. Restoration of postural stability and dynamic balance during gait is essential for maintaining functional independence and reducing fall risk [25, 41]. To assess postural control and gait coordination, we recorded rats as they walked on a narrow beam at baseline, at 18 dpi and at 30 dpi (Fig. 2A). At disease peak (18 dpi), both sham- and ActIIR-AB treated rats showed impaired balance with a significantly increased number of footslips. By 30 dpi, ActIIR-treated, but not the sham-treated rats, returned to baseline footslip count (Fig. 2B). Analysis of the step cycle structure revealed that both groups exhibited prolonged stance (foot on the ground) duration at disease peak (18 dpi) (Fig. 2C), with the ratio of swing (foot in the air) to stance consequently reduced (Fig. 2D). EAN-affected rats likely spend more time with their paws on the ground as a compensatory adaptation to achieve better postural support. By 30 dpi, stance duration and swing to stance ratio reverted to baseline values in ActIIR- but not sham-treated rats (Fig. 2, C and D), showing distinct intra-group recovery trajectories.

**Fig. 3:**
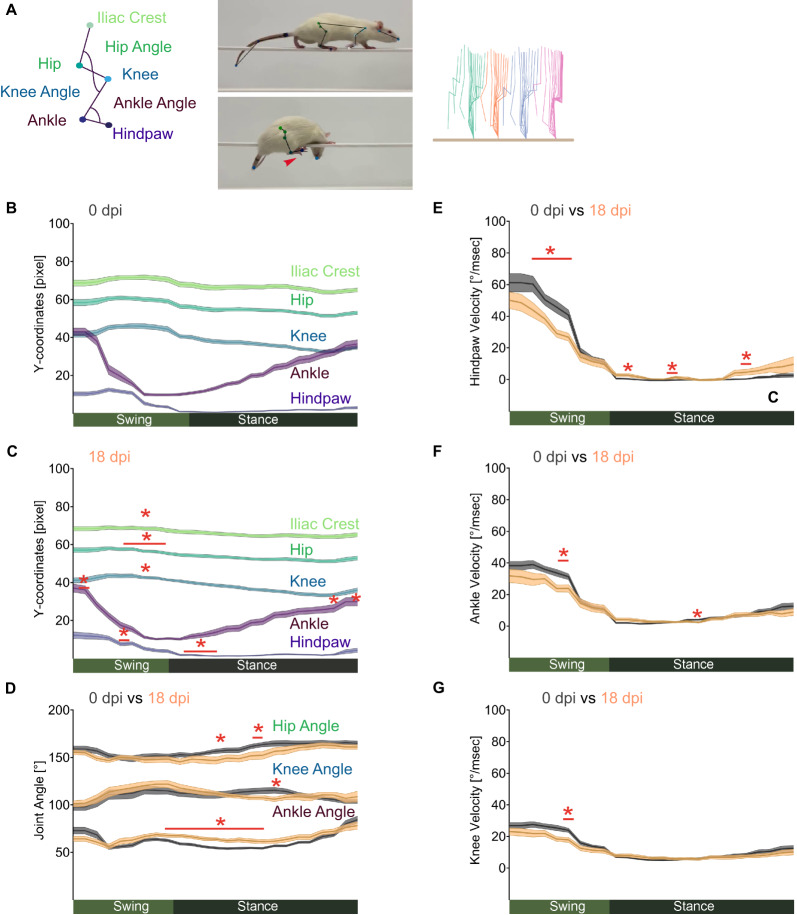
Joint kinematic adjustments underlying postural adaptation in EAN. **A** Exemplary markerless joint tracking of rats using DeepLabCut and a stick diagram of hindlimb joints during a step cycle. To assess EAN-induced gait changes, kinematic features of baseline (0 dpi) and at EAN disease peak (18 dpi) were compared. Joint y-coordinates at baseline (0 dpi) are shown in (**B**) and at EAN disease peak (18 dpi) in (**C**), normalized for differences in body height. The angles of the hindlimb joints for both 0 dpi (dark grey) and 18 dpi (orange) are shown in (**D**) and the comparisons of the lower hindlimb joint velocities of hindpaw in (**E**), ankle in (**F**) and knee in (**G**). In (**B**–**F**), normalized kinematic step cycle analysis using DeepLabCut and AutoGaitA was performed on a total of n = 16 rats at 0 dpi and 18 dpi (EAN induced in two independent experiments with n = 8 per group each). Significant differences between the groups are highlighted with a red beam and their p-values with an asterisk. Data are plotted as mean (darker lines) ± SEM (shaded areas). Statistical significance was calculated with a repeated measure two-way ANOVA by Tukey’s post-hoc test. Details of two-way ANOVA multiple-comparisons are given in the Supplemental Table 2 and 3. *P < 0.05

To better understand how the EAN-induced postural instability affects joint movement and coordination, we used DeepLabCut, a machine-learning-based algorithm [56], to track key body landmarks as the rats crossed a narrow beam (Fig. 3A). Tracked coordinates were analyzed using AutoGaitA to compare changes in limb kinematics across groups [33]. First, we characterized how EAN affects kinematic features during walking. At EAN disease peak (18 dpi), height (y-coordinates) of the joints was more elevated compared to baseline (0 dpi, Fig. 3B), likely due to the physiological rat body growth over 18 days (Supplemental Fig. 2A, and Supplemental Table 2). Therefore, to enable more accurate hindlimb joint comparisons, we normalized hindlimb joints for height differences (Fig. 3C). EAN induced a lowering of the hindlimb mostly during swing (Fig. 3, B and C, and Supplemental Table 3). At 18 dpi, joint angles were not affected during swing, but body postures were supported during stance by elongating the distal part of the limb (extended ankle angle) and crouching the proximal part (hip and knee slightly more flexed) (Fig. 3D, and Supplemental Table 3). Joint movement velocities were slightly reduced during the swing phase (Fig. 3, E–G, Supplemental Fig. 2, B and C, and Supplemental Table 3). Taken together, the joint kinematic alterations observed at the peak of EAN indicate that the rats adopt compensatory strategies to stabilize posture. During swing, they restrict limb elevation (lower joint heights) and reduce velocity to improve the precision of paw placement. During stance, they adjust the coordination of distal and proximal limb extension to enhance body stabilization while balancing on the beam.

**Fig. 4:**
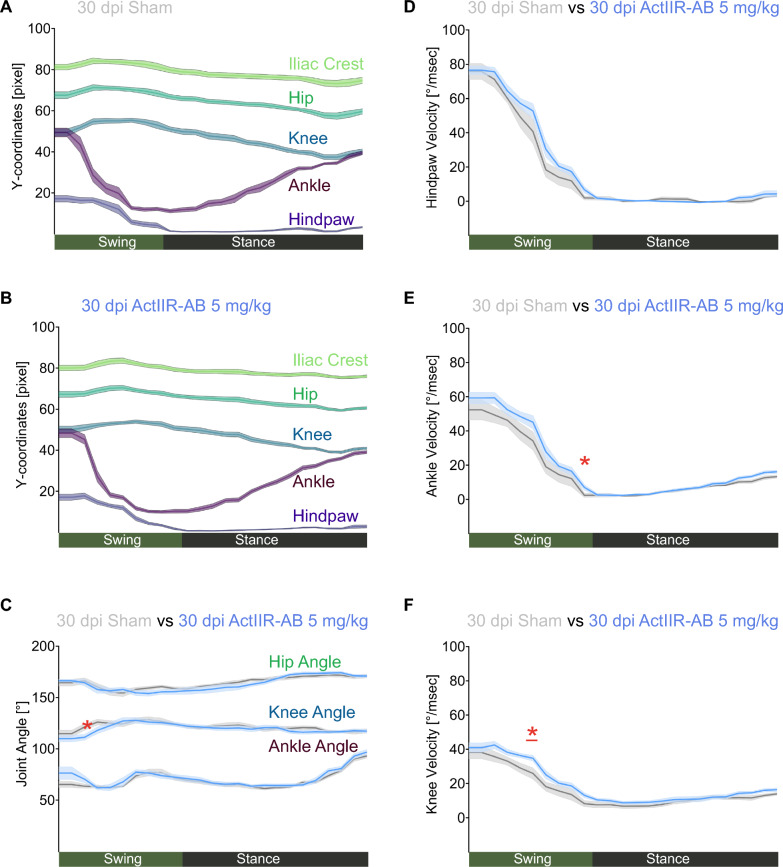
Modest residual kinematic adaptations reflect incomplete postural recovery. No differences for the Y-coordinates at 30 dpi (days post immunization) were seen between sham- (**A**) and ActIIR-AB-treatment (**B**). Joint angles at 30 dpi for sham- (light grey) and ActIIR-AB-treated animals (blue) are shown in (**C**). Joint velocities of hindpaw (**D**), ankle (**E**) and knee (**F**) are shown at 30 dpi for the sham- (light grey) versus the ActIIR-AB treated animals (blue). In (**A**–**F)**, normalized kinematic step cycle analysis using DeepLabCut and AutoGaitA was performed on n = 8 rats per group (ActIIR-AB treatment (5 mg/kg) and sham-treatment) at the same timepoint (30 dpi). Significant differences between the groups are highlighted with a red beam and their p-values with an asterisk. Data are plotted as mean (darker lines) ± SEM (shaded areas). Statistical significance was calculated with a mixed two-way ANOVA (for sham vs ActIIR-AB) followed by Tukey’s post-hoc test. Details of two-way ANOVA multiple-comparisons are given in the Supplemental Table 4. *P < 0.05

Next, to assess whether ActIIR-AB treatment affected these kinematic adaptations, we analyzed sham- and ActIIR-AB-treated rats at the end of the recovery phase (30 dpi), observing only minor differences between groups (Fig. 4, A–C, and Supplemental Table 4). In contrast to ActIIR-AB-treated rats, the joint velocities of ankle, knee and hip of sham-treated rats were mildly reduced during swing, suggesting that sham-treated rats might still rely on slow paw placement to adapt to EAN-induced postural instability (Fig. 4, D–F, and Supplemental Fig. 2, D, and E, and Supplemental Table 4).

### ActIIR inhibition improves recovery without detectable changes in nerve immune markers or myelination

ActIIRs are broadly expressed across the body, including immune cells [53, 63], and have been implicated in several immunoregulatory processes, such as the (auto-)regulation of macrophages and T cell responses [26, 34, 87, 99], with both cell types contributing to EAN and GBS pathophysiology [31, 39]. In addition, ActIIR signaling might promote Schwann cell proliferation, possibly modulating (re-)myelination [52]. Notably, we observed an improved recovery of MNCV after ActIIR-AB treatment (Fig. 1, E–G). To determine the mechanisms underlying ActIIR-AB-mediated rescue of EAN symptoms, we examined the anatomical and inflammatory changes in the sciatic nerves at 30 dpi. ActIIR-AB treatment did not affect the number of T cells (Supplemental Fig. 3, A–C) and macrophages (Supplemental Fig. 3, D-F) infiltrating the sciatic nerve. Real-time PCR analysis of the sciatic nerve tissue showed no differences in pro- (IL-6, IL-1β, TNF-α) (Supplemental Fig. 4, A–C) and anti-inflammatory cytokines (IL-4, IL-10) (Supplemental Fig. 4, D and E). Immunohistochemical analysis revealed no differences in myelinated fiber counts (Supplemental Fig. 5, A–C) or semi-quantitative Fluoromyelin staining (Supplemental Fig. 5, D–F). Taken together, ActIIR inhibition improved functional recovery without detectable modulation of peripheral nerve inflammation or myelination markers at 30 dpi, consistent with a mechanism acting downstream of inflammation-driven nerve injury.

**Fig. 5:**
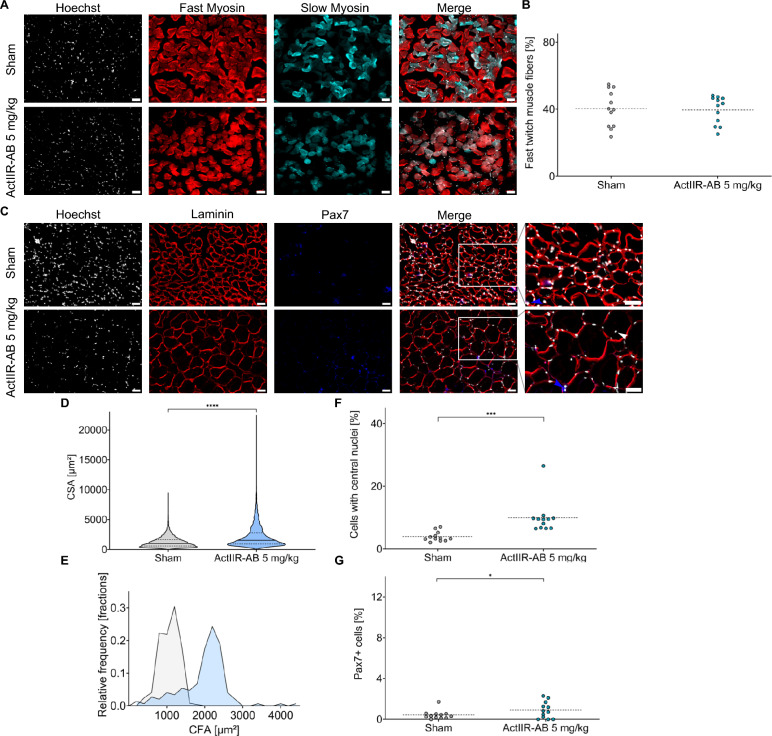
ActIIR inhibition preserves muscle fiber size during the recovery of EAN. **A** Representative immunostaining for fast and slow twitching muscle fibers of the tibialis anterior muscles of a sham- and an anti-ActIIR-AB-treated rat. Scale bar: 50 µm. **B** Nested analysis of the percentage of fast twitching muscle fibers of the tibialis anterior muscle. Overall, n = 116 images for the sham-treated rats and n = 111 images of the treatment group were analyzed (n = 12 rats per group). **C** Representative immunostainings of the tibialis anterior muscles at 30 dpi of sham- and anti-ActIIR-AB-treated rats for Laminin, Pax7 and Hoechst. Scale bar: 50 µm. The white arrows indicate exemplary central nuclei in the center of a muscle fiber. Exemplary Pax7 expressing muscle cells are highlighted with a blue arrow. **D** Cross-sectional area of the muscle fibers (CSA) plotted as truncated violin plot showing the median (solid line) and the quartiles (dashed lines). **E** Histogram depicting the relative frequency distribution of the CSA fractions. A right shift toward increased muscle fiber size is observed for the treatment group. **F** Nested analysis of the percentage of muscle cells with central nuclei and Pax7 expressing muscle fibers (**G**) as markers of muscle hypertrophy. For quantification of (**D, E and F, G**), 10 images per muscle of n = 12 rats per group were taken randomly at a 20 × magnification. Overall, n = 14,100 muscle fibers for the sham-treated rats and n = 8263 muscle fibers of the treatment group were analyzed. An unpaired two-tailed Mann–Whitney test was used for (**D**). A nested t-test was applied for (**B, F, and G**), with data plotted as the overall mean (dashed line), and the mean of each technical replicate as a dot. *P < 0.05, ***P < 0.001, ****P < 0.0001

### ActIIR inhibition preserves muscle fiber size

ActIIR blockade has been shown to not only prevent muscle atrophy but to also induce hypertrophy [44]. To determine whether hypertrophy contributes to the observed improved motor recovery in EAN, we analyzed the muscle fiber morphology in the tibialis anterior, a commonly affected muscle in both EAN and GBS [1, 38, 85] at 30 dpi. Fiber type composition (fast- versus slow-twitch) was unchanged between groups. However, fast-twitch fibers in sham-treated muscles appeared smaller and more heterogeneous, indicative of reduced fiber caliber (Fig. 5, A and B, and Supplemental Fig. 6A). Laminin staining identifying the contour of individual muscle fibers showed a significantly larger cross-sectional area of muscle fibers in ActIIR-AB-treated rats (Fig. 5, C and D). This was confirmed by Feret’s diameter analysis (Supplemental Fig. 6B), resulting in a rightward shift towards larger fiber diameters (Fig. 5E, Supplemental Fig. 6C). Notably, we also observed a significant increase in centrally located nuclei (Fig. 5, C and F, and Supplemental Fig. 6D) and Pax7 expressing satellite cells (Fig. 5, C and G, and, Supplemental Fig. 6E), both markers of active myogenesis [68, 84].

**Fig. 6:**
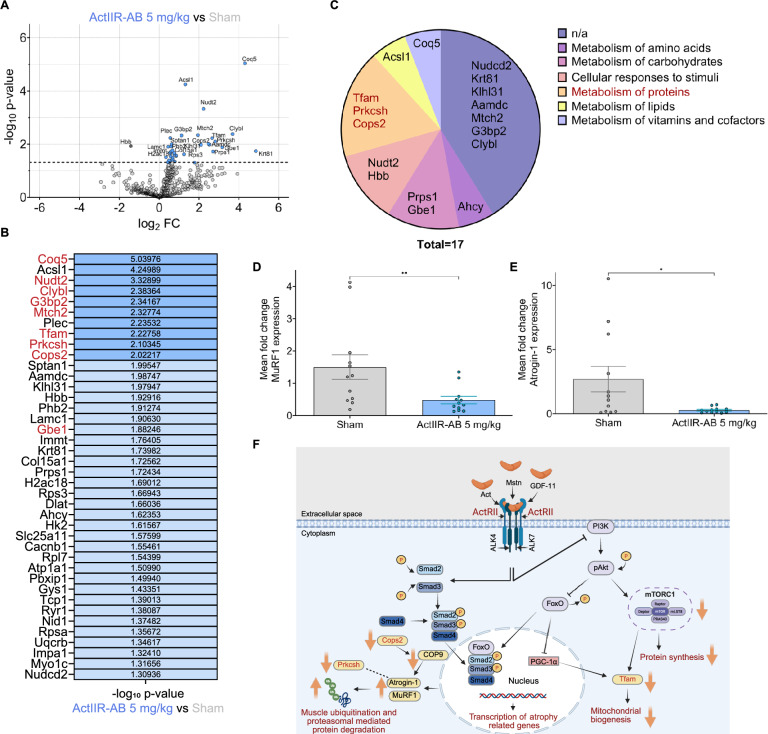
ActIIR inhibition reduces expression of MuRF1 and Atrogin-1 in skeletal muscle after EAN. **A** Volcano plot of differentially expressed proteins in tibialis anterior muscles of 5 mg/kg anti-ActIIR-AB- versus sham-treated rats at 30 dpi. Blue colored circles mark significantly upregulated proteins in the ActIIR-AB-treated rats at *P* < 0.05. The significantly downregulated protein (Hbb) is marked by a dark grey circle. N = 4 rats per group were used. **B** A heat map of the differentially expressed proteins of anti-ActIIR-AB treated animals, sorted by − log_10_ *p*-value. Proteins highlighted in red in the heat map fulfill stringent FOXO/SMAD chromatin annotation criteria, including conserved promoter-proximal (≤ 1 kb from the transcription start site) binding by at least one FOXO family member (FOXO1, FOXO3, or FOXO4) and by all three canonical SMAD transcription factors (SMAD2, SMAD3, and SMAD4) in both mouse and human chromatin ChIP-Atlas datasets, filtered for muscle- and lineage-relevant chromatin immunoprecipitation experiments. This annotation provides contextual support for FOXO/SMAD pathway involvement and does not imply direct transcriptional regulation in the rat model. **C** Reactome platform analysis of the top hits (*p* < 0.05 and log_2_(fold change (FC)) of < −1, < 1) for enriched broader biological pathways, showing three proteins that are involved in protein metabolism in red. Proteins without further pathway information for the organism *Rattus norvegicus* were marked with n/a. **D** Quantitative real-time PCR for expression of MuRF1 and for Atrogin-1 in (**E**). **F** Converging pathway of ActIIR-binding in muscle cells inducing muscle atrophy. Created with biorender.com. Data are plotted as mean ± SEM. An unpaired two-tailed Mann–Whitney test was used for (**D**) and (**E**). Student’s t test was used to test for significant (*p* < 0.05) changes in protein abundance

### ActIIR inhibition is associated with reduced activation of muscle atrophy-related FoxO-dependent programs

Different Smad proteins have distinct effects on muscle fiber size and are differentially activated depending on the ligand-specific ActIIR signaling pathway: Smads1/5/8 stimulate the AKT-mTOR axis to promote muscle hypertrophy, whereas Smads2/3 suppress AKT-mTOR signaling to drive atrophy [28, 81, 83]. The latter pathway requires FoxO-mediated activation of the ubiquitin–proteasome pathway, ultimately inducing expression of the E3 ubiquitin ligases MuRF1 and Atrogin-1 [5, 6, 80]. To distinguish the differential contribution of muscle hypertrophy and muscle fiber preservation, we performed proteomic analysis of the tibialis anterior at 30 dpi (n = 4 per group). Samples from rats at the peak of EAN (18 dpi) served as an additional control (n = 3). The majority (97.5%) of the 40 (5.2%) differentially expressed proteins were upregulated in ActIIR-treated animals at 30 dpi (Fig. 6, A, and B). STRING and WikiPathways proteome network analysis only showed enrichment of pathways involved in striated muscle contraction (Supplemental Fig. 7A). Almost no differences were seen between the tibialis anterior proteome at 18 dpi versus 30 dpi (ActIIR-AB vs. 18 dpi: 6 out of 762 proteins differentially expressed, 0.8%; sham vs. 18 dpi: 8 out of 762, 1%; Supplemental Fig. 7, B and C). Clustering of the 17 top hits in the muscle proteome at 30 dpi (log_2_FC > 1 or < − 1, *p* < 0.05) using the Reactome platform [61] revealed that only three proteins—Tfam, Prkcsh and Cops2—are part of a small cluster within the broad category “metabolism of proteins” (Fig. 6C, and Supplemental Table 5). Notably, all these proteins are linked to muscle atrophy via the FoxO-ubiquitin–proteasome cascade. FoxO, the major transcriptional activator of ubiquitin-mediated proteolysis via Atrogin-1 and MuRF1, has been shown to decrease Tfam, thereby reducing mitochondrial biogenesis [12, 79]. Prkcsh has been identified as an Atrogin-1 targeted protein during myostatin-induced muscle atrophy [54]. Cops2 is a core subunit of the COP9 signalosome, which regulates cullin deneddylation, thereby modulating the activity of Skp1-Cullin1-F-box E3 ligase complexes, including those that incorporate the F-box protein Atrogin-1 [14, 27].

Because proteomic profiling alone does not provide information on upstream transcriptional regulation, we next assessed whether the proteins regulated by ActIIR inhibition are compatible with regulation by the FOXO/SMAD transcriptional axis. The 17 top-hit proteins were mapped to publicly available chromatin immunoprecipitation datasets using ChIP-Atlas 3.0 [100] to provide contextual support for pathway involvement. As muscle-specific chromatin immunoprecipitation datasets for rat are sparse and FOXO/SMAD signaling pathways are highly conserved across species, mouse and human datasets were leveraged using ortholog mapping. Nine of the 17 proteins (including Tfam, Prkcsh und Cops2) fulfilled stringent filtering criteria, including conserved promoter-proximal binding (≤ 1 kb from the transcription start site) by at least one FOXO family member and by all three canonical SMAD transcription factors (SMAD2, SMAD3, and SMAD4) in both mouse and human datasets. This conservative annotation is consistent with involvement of FOXO/SMAD-associated transcriptional programs in the proteomic response to ActIIR inhibition.

At the RNA-level, quantitative PCR analysis of ActIIR-AB-treated animals versus sham-treated rats at day 30 dpi confirmed significantly reduced expression of Atrogin-1 and MuRF1, downstream targets of FoxO (Fig. 6, D and E). Together, these proteomic and transcriptomic data are consistent with attenuation of FoxO-dependent atrophy programs in skeletal muscle after ActIIR inhibition (Fig. 6F).

## Discussion

The myostatin signaling pathway has garnered significant attention in recent years, but despite compelling preclinical data, clinical translation has proven challenging. Early trials targeting this pathway to counter muscle wasting have shown limited efficacy, likely due to complex ligand-receptor redundancy within the TGF-β superfamily [44, 45]. More encouraging results have emerged from studies employing greater target specificity, e.g., the positive Phase II results from the TOPAZ trial evaluating Apitegromab (a promyostatin inhibitor) in patients with spinal muscular atrophy [15], or in studies repurposing drugs like Bimagrumab [32].

Our work extends the therapeutic scope of ActIIR inhibition to secondary neurogenic muscle atrophy in EAN, an animal model of GBS, the most common acute immune-mediated neuropathy [49]. We hypothesized that delaying or limiting secondary muscle atrophy until muscle reinnervation or until the resolution of the nerve conduction block could promote motor recovery. Our data show that ActIIR inhibition during the recovery phase enhanced motor performance in a dose-dependent manner in EAN. The improved functional recovery in ActIIR-AB-treated rats was confirmed by our multimodal approach, including neuritis scores, grip strength (Fig. 1) and kinematic gait analysis (Figs. 2–4, and Supplemental Fig. 2). Importantly, the simple kinematic analysis set-up, built by combining a commercially available phone camera, AI-based markerless tracking and a standardized framework for gait analysis, holds high translational value for assessing and comparing gait dysfunction and recovery in GBS patients and animal models [18, 33, 98].

Several studies have shown that the immunological peripheral nerve microenvironment influences the functional and histological recovery in EAN and peripheral nerve injuries [11, 55, 86]. The myostatin pathway potentially affects the immune environment, given that both innate and adaptive immune cells can express ActIIRs and can produce Activin A [26, 50, 96]. However, at the end of the recovery phase (30 dpi), ActIIR inhibition did not detectably alter sciatic nerve immune cell infiltration or inflammatory cytokine transcript levels (Supplemental Figs. 3 and 4). These data indicate that secondary muscle atrophy represents a recovery-limiting pathological process downstream of inflammatory nerve injury, and suggest that motor outcome after immune-mediated neuropathy may be improved by targeting this process.

ActIIR-AB-treated rats showed significantly larger cross-sectional muscle fiber area compared to sham controls (Fig. 5, A–E, and Supplemental Fig. 6, B and C). This improvement may reflect ActIIR inhibition either in limiting secondary muscle atrophy or in inducing hypertrophy in muscle fibers not affected by EAN. Prevention of muscle atrophy and muscle hypertrophy can both result from ActIIR blockade, primarily through inhibition of Activin A- and Myostatin-driven Smad2/3 signaling [81]. Smad1/5/8 signaling acts in parallel to support hypertrophic growth and can be initiated by binding of Bone Morphogenetic Proteins (BMPs) to ActIIRs via AKT-mTOR [83, 95]. In contrast, Smad2/3 signaling, initiated by Activin A and Myostatin, induces atrophy via FoxO-mediated activation of the ubiquitin–proteasome axis, leading to increased expression of the E3 ligases MuRF1 and Atrogin-1 [28, 80, 81]. The translational relevance of this differential role of Smad signaling was underscored by the discontinued phase II trial of Ramatercept (ACE-031) in Duchenne muscular dystrophy. Ramatercept, a ligand trap for ActIIRB, binds myostatin and other related ligands, including BMP9. The sequestration of BMP9 has been linked to reduced endothelial ALK1- Smad1/5/8 signaling and to unwanted adverse effects in the study, including epistaxis and telangiectasias [10, 17, 51, 89]. Notably, impaired endothelial ALK1-Smad1/5/8 signaling is also observed in hereditary hemorrhagic telangiectasia [67, 77]. This limitation led to the development of a heterodimeric ligand trap, combining ALK4- and ActIIRB-domains, which selectively sequesters Smad2/3-activating ligands while sparing BMP9 and Smad1/5/8 signaling. This ligand trap approach already showed promising results in mouse models for Duchenne muscular dystrophy and for secondary neurogenic muscle atrophy, amyotrophic lateral sclerosis [51].

Mechanistically, our data suggest that the observed motor improvements are associated with preserved muscle fiber size and reduced expression of FoxO-dependent muscle atrophy-related programs. This conclusion is supported by significantly reduced expression of Atrogin-1 and MuRF1 in the tibialis anterior, a commonly affected muscle in both EAN and GBS (Fig. 6) [1, 38, 85]. Nevertheless, we noted an increase in markers of myogenesis and hypertrophy, including elevated Pax7 expressing satellite cells and a higher prevalence of centrally located nuclei in muscle fibers (Fig. 5, F and G, Supplemental Fig. 6, D and E). This could be a consequence of the competitive interaction between the Smad1/5/8 and Smad2/3 signaling pathways. In particular, the two pathways rely on the shared co-Smad4, which is essential for the formation of an active transcription complex and consequently transcriptional activity [83, 91, 97]. Therefore, increased signaling through one pathway may limit Smad4 availability for the other. In the context of ActIIR inhibition, this would entail reduced Smad2/3 signaling and fewer Smad2/3-Smad4 complexes, potentially increasing Smad4 availability for BMP-activated Smad1/5/8 signaling and thereby favoring anabolic transcriptional programs. However, direct evidence that Smad4 availability is limiting and redistributes between these complexes in skeletal muscle is currently lacking. This hypothesis may explain the dual effects we observed: limitation of atrophy alongside modest hypertrophic changes. This may also explain the striking effects of ActIIR inhibition on muscle hypertrophy in several animal models and human trials [43, 45, 46, 65, 71]. Notably, histological evidence of muscle atrophy in mice and humans after denervation is observed 10–21 days after denervation, while neuronal atrophy-induced myogenesis commences within the first 1–2 months of the post-denervation phase [2, 7, 51].

Initiating treatment at the onset of the recovery phase reflects a clinically relevant paradigm, as current first-line immunotherapies (intravenous immunoglobulins or plasmapheresis) are typically administered during the acute effector phase and may interfere with the pharmacokinetics or bioavailability of co-administered biologics such as monoclonal antibodies [20, 49]. Our approach of augmenting post-inflammatory functional recovery targets a distinct therapeutic window and provides a synergistic strategy to treat secondary neurogenic muscle atrophy.

Our study has limitations. First, the involvement of the human ActIIR-pathway in secondary muscle atrophy induced by immune neuropathies remains to be further explored as well as potential long-term side effects of systemic ActIIR inhibition. A further limitation is the lack of quantitative assessment of axonal degeneration and reinnervation using ultrastructural morphometry at 18 and 30 dpi. However, our behavioral, electrophysiological and histological data showed a comparable disease severity at EAN disease peak without effects of ActIIR inhibition on myelination at the end of the recovery phase. Classical myogenic differentiation markers were not assessed, limiting definitive discrimination between muscle regeneration and adaptive remodeling. Lastly, as changes in muscle fiber size may result from increased protein synthesis, reduced autophagic flux, and/or decreased protein degradation via ubiquitin–proteasome pathways, more comprehensive proteomic analyses combined with poly-ubiquitination profiling are required to fully elucidate how ActIIR inhibition modulates FoxO-associated protein turnover and the regulation of muscle atrophy in immune neuropathies.

## Conclusions

In conclusion, our findings indicate that ActIIR-AB administered during the recovery phase enhances motor recovery in EAN and preserves muscle fibers, with no detectable effects on peripheral nerve inflammation or remyelination at the end of the recovery phase. These data suggest that muscle preservation strategies may offer a valuable adjunctive approach for improving long-term outcomes in patients recovering from immune neuropathies such as GBS, shifting part of the therapeutic focus from nerve to muscle. Importantly, the therapeutic timing of targeting the recovery phase avoids interference with acute immunotherapies and enables sequential treatment regimens. Future studies are needed to explore the translational potential of these findings in clinical settings and delineate the molecular interplay between Smad signaling pathways during denervation and reinnervation processes.

## Supplementary Information

Supplementary Material 1.

Supplementary Material 2.

Supplementary Material 3.

Supplementary Material 4.

Supplementary Material 5.

Supplementary Material 6.

Supplementary Material 7.

Supplementary Material 8.

Supplementary Material 9.

Supplementary Material 10.

Supplementary Material 11.

Supplementary Material 12.

Supplementary Material 13.

## Data Availability

The datasets supporting the conclusions of this article are included within the article and its additional files. Additional data are available from the corresponding author upon reasonable request. The mass spectrometry proteomics data are available under accession number PXD070456 in the ProteomeXchange Consortium.

## Abbreviations

AB: Antibody
ActIIR: Activin type II receptors
BMP: Bone Morphogenetic Protein
CIDP: Chronic inflammatory demyelinating polyneuropathy
CMAP: Compound muscle action potential
DPI: Days post immunization
EAN: Experimental autoimmune neuritis
GBS: Guillain-Barré syndrome
MNCV: Motor nerve conduction velocity
