# Supplementary figures and images for: ActIIR inhibition improves motor outcome and preserves muscle fibers after experimental autoimmune neuritis
Source: Acta Neuropathol Commun. 2026 Mar 27;14:86. doi: 10.1186/s40478-026-02277-z (PMC13063503; doi:10.1186/s40478-026-02277-z)

**A**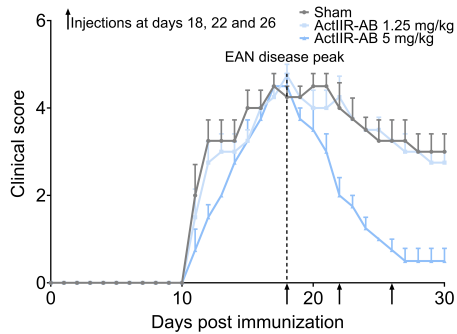**B**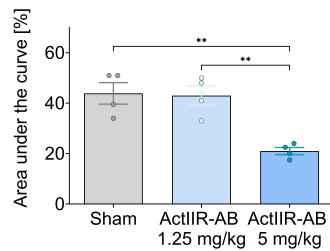**C**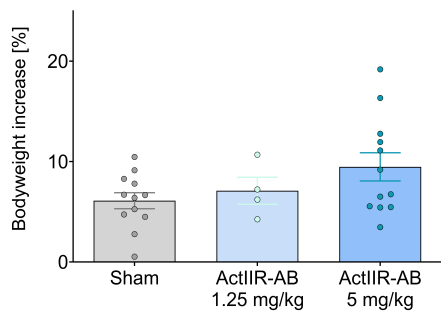**D**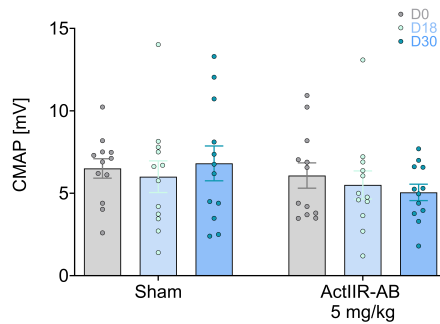

Supplement: Supplementary file 1 — Supplementary Material 1. [file 40478_2026_2277_MOESM1_ESM.pdf]

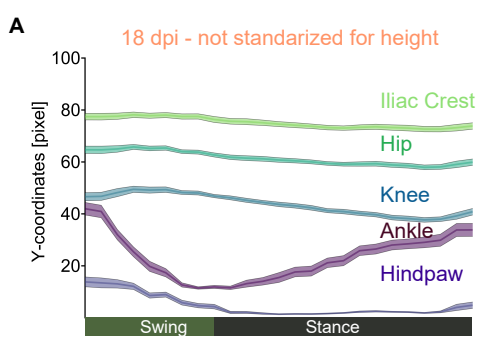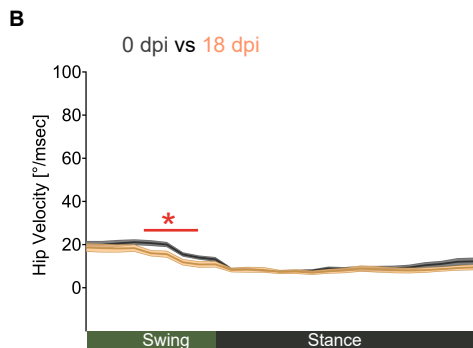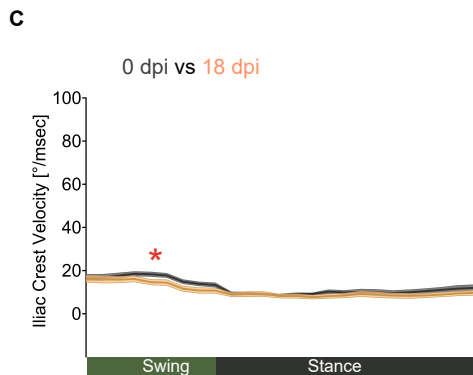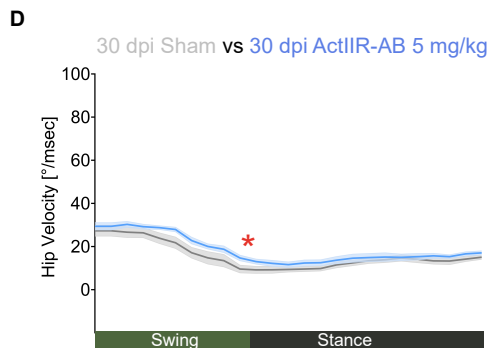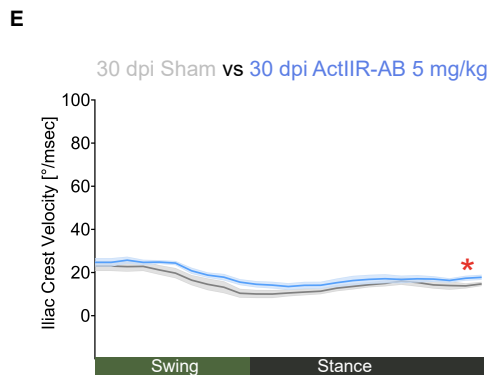

Supplement: Supplementary file 2 — Supplementary Material 2. [file 40478_2026_2277_MOESM2_ESM.pdf]

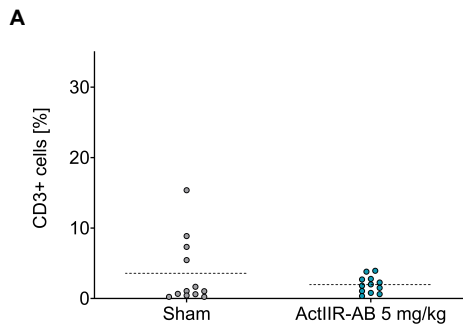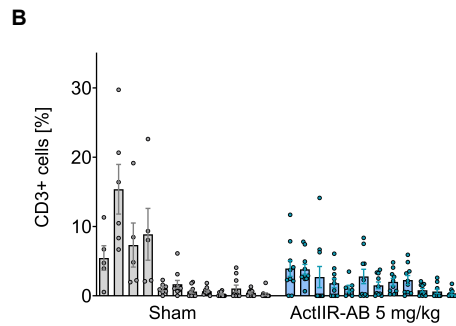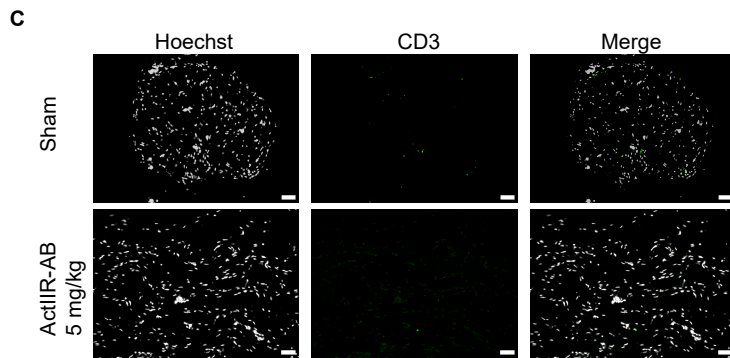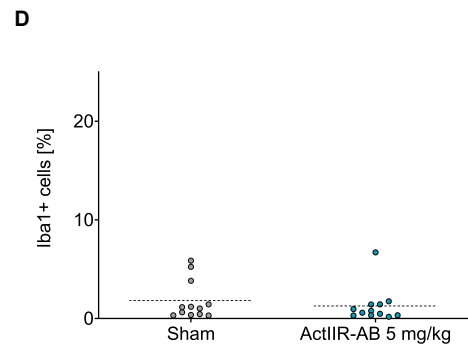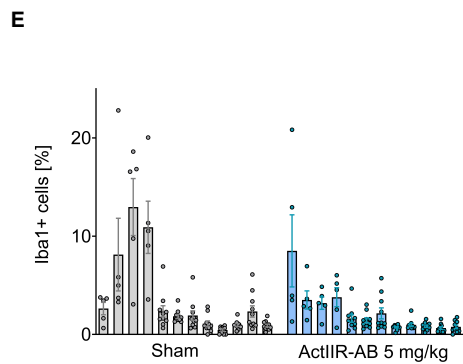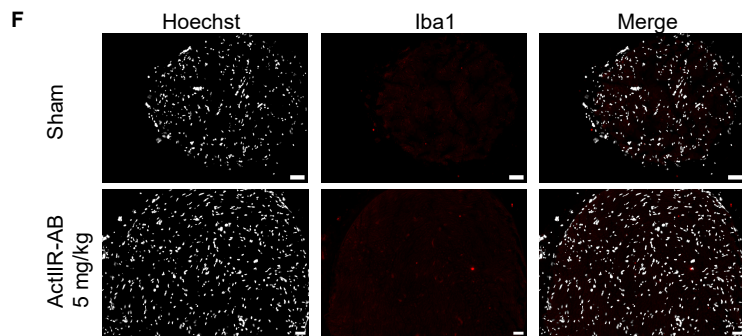

Supplement: Supplementary file 3 — Supplementary Material 3. [file 40478_2026_2277_MOESM3_ESM.pdf]

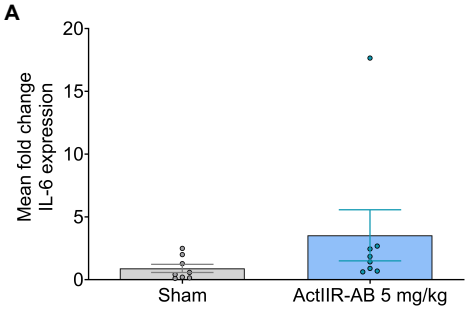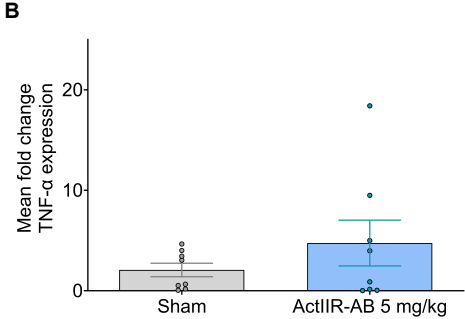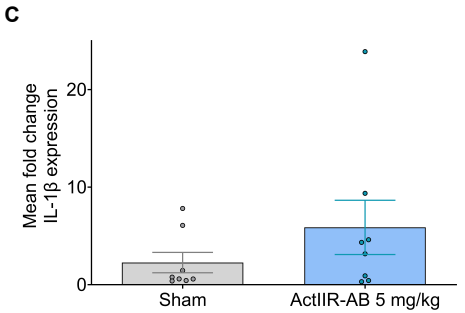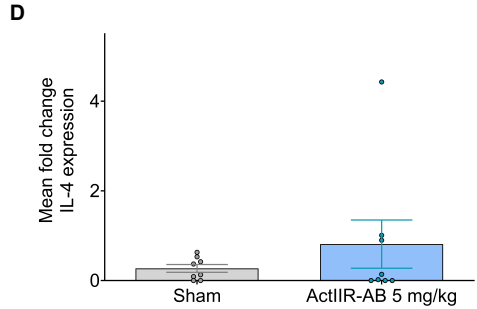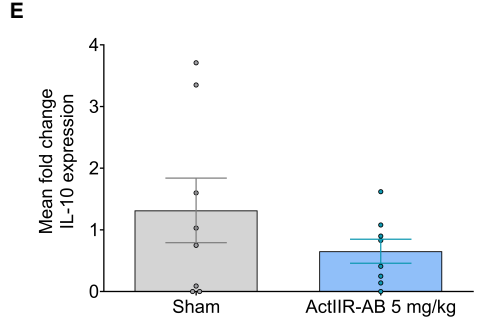

Supplement: Supplementary file 4 — Supplementary Material 4. [file 40478_2026_2277_MOESM4_ESM.pdf]

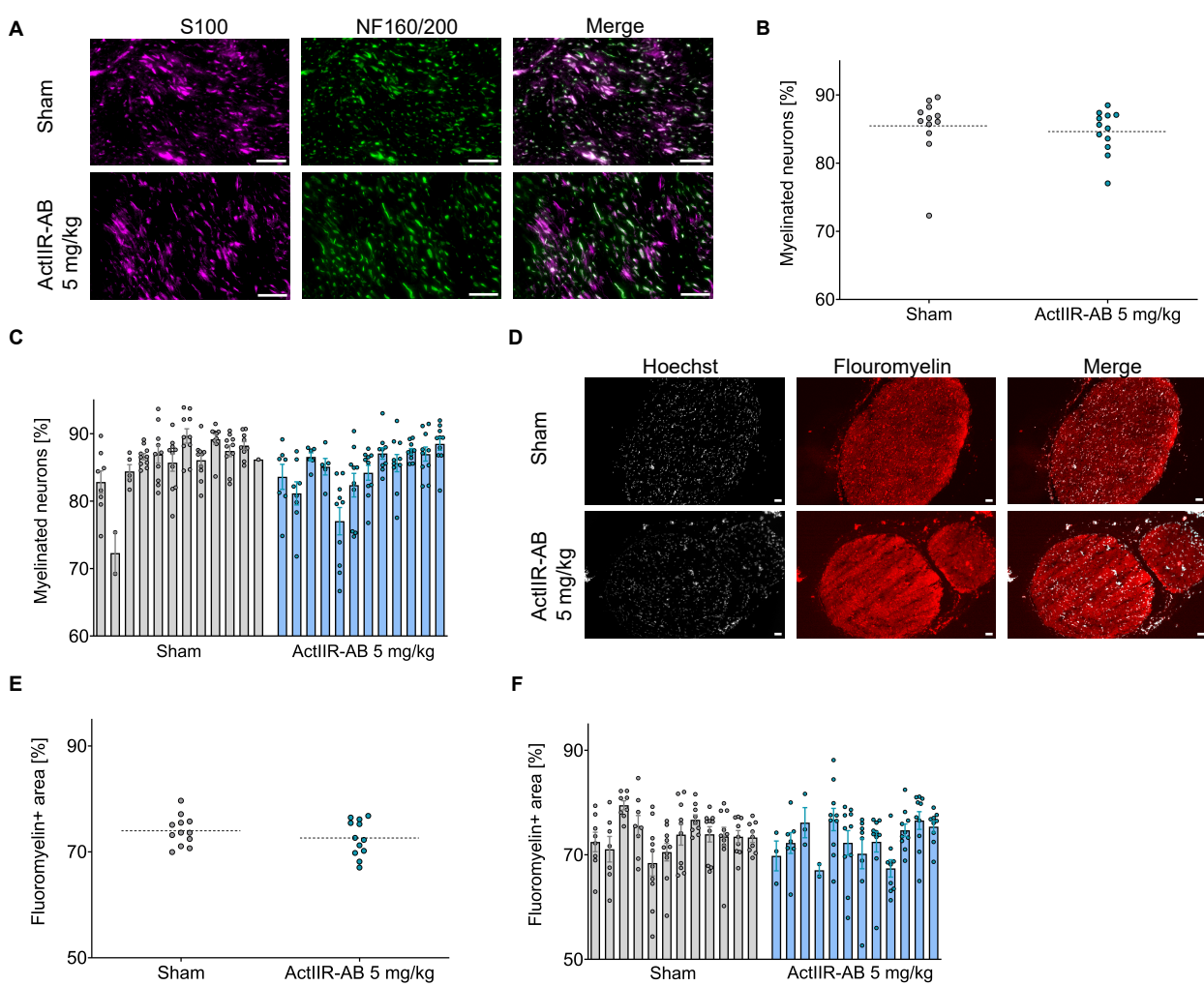

Supplement: Supplementary file 5 — Supplementary Material 5. [file 40478_2026_2277_MOESM5_ESM.pdf]

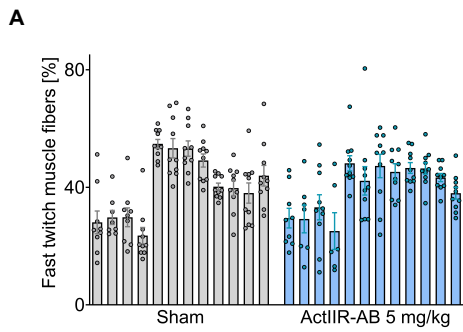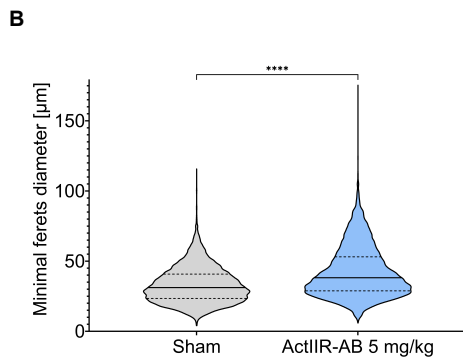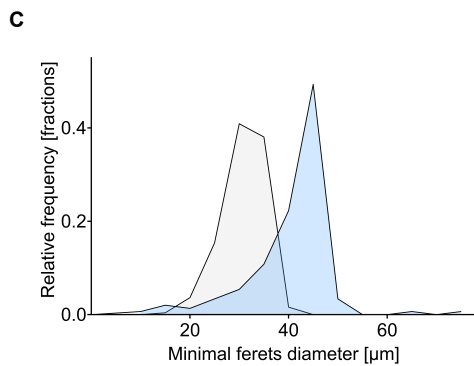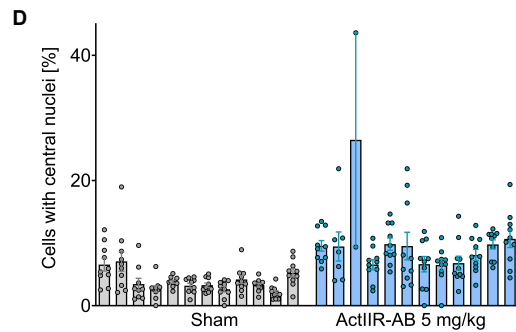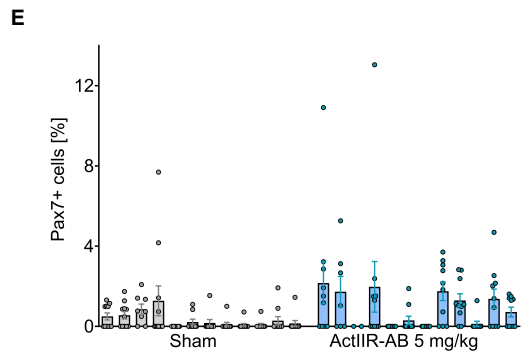

Supplement: Supplementary file 6 — Supplementary Material 6. [file 40478_2026_2277_MOESM6_ESM.pdf]

**A****WikiPathways enrichment**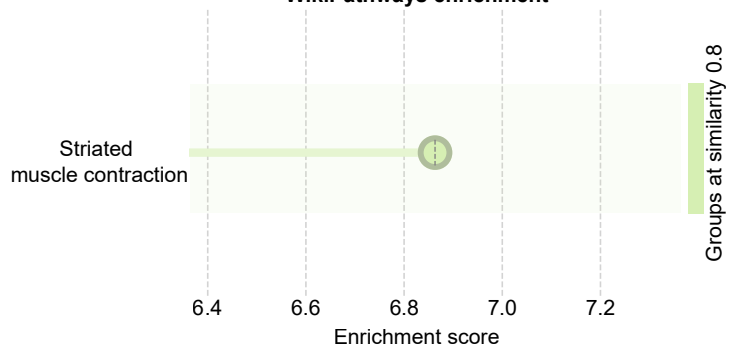**B****ActIIIR-AB 5 mg/kg vs 18 dpi**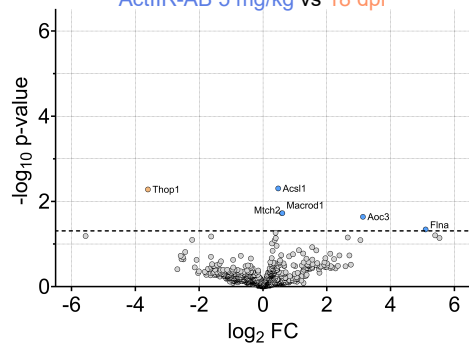**C****Sham vs 18 dpi**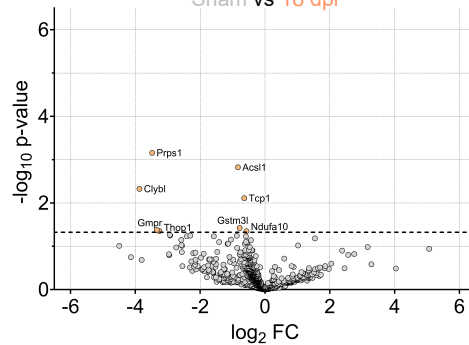

Supplement: Supplementary file 7 — Supplementary Material 7. [file 40478_2026_2277_MOESM7_ESM.pdf]
