# Supplementary material for: ActIIR inhibition improves motor outcome and preserves muscle fibers after experimental autoimmune neuritis
Source: Acta Neuropathol Commun. 2026 Mar 27;14:86. doi: 10.1186/s40478-026-02277-z (PMC13063503; doi:10.1186/s40478-026-02277-z)
Supplement: Supplementary file 8 — Supplementary Material 8. [file 40478_2026_2277_MOESM8_ESM.pdf]

Supplemental Table 1. Primers for quantitative real-time PCR.

Primer	Primer sequence	Product length (bp)
MuRF1-fwd	GCCGCTCATCAAGAGCATTG	212
MuRF1-rev	CCTTACCCTCTGTGGACACG	
Atrogin-1-fwd	AGCTTGTGCGATGTTACCCA	142
Atrogin-1-rev	GGTGAAAGTGAGACGGAGCA	
TNF-α-fwd	CATCCGTTCTCTACCCAGCC	146
TNF-α-rev	AATTCTGAGCCCGGAGTTGG	
IL-1ß-fwd	GACTTCACCATGGAACCCGT	120
IL-1ß-rev	GGAGACTGCCCATTCTCGAC	
IL-4-fwd	CAGACGTCCTTACGGCAACA	166
IL-4-rev	AGACCGCTGACACCTCTACA	
IL-6-fwd	CCCAACTTCCAATGCTCTCCT	131
IL-6-rev	AGCACACTAGGTTTGCCGAG	
IL-10-fwd	TCAGCACTGCTATGTTGCCT	237
IL-10-rev	GGCTTGGCAACCCAAGTAAC	
