# Supplementary material for: ActIIR inhibition improves motor outcome and preserves muscle fibers after experimental autoimmune neuritis
Source: Acta Neuropathol Commun. 2026 Mar 27;14:86. doi: 10.1186/s40478-026-02277-z (PMC13063503; doi:10.1186/s40478-026-02277-z)
Supplement: Supplementary file 13 — Supplementary Material 13. [file 40478_2026_2277_MOESM13_ESM.docx]

**SUPPLEMENTAL FIGURE LEGENDS**

**Supplemental Figure 1. Related to Figure 1. Dose titration experiment and supplemental clinical and electrophysiological data.** (**A**) Disease course in the first dose-titration experiment. (**B**) AUC of the neuritis score from 18 to 30 days post immunization (dpi) assessing recovery in the dose-titration groups. (**C**) shows the percent body weight change during the recovery phase from 18 to 30 dpi. (**D**) Compound muscle action potential (CMAP) of dorsal foot muscles after distal stimulation of the sciatic nerve. In the dose-titration experiment (**A** and **B**), n=4 animals per group were used. Body weight change and electrophysiological data are shown as pooled data for all experiments (n=12 animals for sham and high dosage of 5 mg/kg ActIIR-AB, n=4 for 1.25 mg/kg ActIIR-AB for body weight change). Data are plotted as mean ± SEM. Differences between three or more groups were tested by one-factor analysis of variance (ANOVA) followed by the Holm-Sidak multiple-comparison test. **P < 0.01.

**Supplemental Figure 2. Related to Figure 4. Additional kinematic gait data.** (**A**) Y-coordinates at 18 dpi (days post immunization) without height standardization. Comparisons of the joint velocities at baseline (0 dpi, orange) and EAN disease peak (18 dpi, dark grey) are shown for hip (**B**) and iliac crest (**C**). To assess any treatment effects at the end of EAN recovery (30 dpi), the hip (**D**) and iliac crest (**E**) joint velocities of sham- (light grey) and 5 mg/kg ActIIR-AB-treated rats were (blue) compared. In (**B**) and (**C**), kinematic step cycle analysis using DeepLabCut and AutoGaitA was performed on n=16 rats per timepoint (EAN induced in two independent experiments with n=8 per group each), while in (**D**) and (**E**) n=8 rats per group at the same timepoint (30 dpi) are shown (EAN was induced in two independent experiments). Significant differences between the groups are highlighted with a red beam and their p-values with an asterisk. Data are plotted as mean (darker lines) ± SEM (shaded areas). Statistical significance was calculated with either a repeated measure two-way ANOVA (for 0 dpi vs 18 dpi) or mixed two-way ANOVA (for sham vs ActIIR-AB) followed by Tukey’s post-hoc test. Details of two-way ANOVA multiple-comparisons are given in the Supplemental Tables 2-4. *P < 0.05.

**Supplement Figure 3**. **ActIIR inhibition does not alter sciatic nerve immune cell infiltration**. (**A**) and (**B**): Nested analysis of CD3+ cells (sham: n=98, 5 mg/kg anti-ActIIR-AB: n=117 quantified images). (**C**) Immunostaining for CD3+ T-cells of the sciatic nerve and Hoechst. Scale bar: 50 µm. (**D**) and (**E**): Nested analysis of Iba1+ cells of the sciatic nerve. N=100 images for both groups were quantified. (Representative immunostaining for Iba1+ macrophages and Hoechst of the sciatic nerve is shown in (**F**). Scale bar: 50 µm. For quantifications of (**A**), (**B**) and (**D**), (**E**), 5-10 images of n=12 rats per group from distal and proximal nerve stumps were taken randomly at a 40x magnification. A nested t-test was applied, with data plotted as the overall mean (dashed line), and the mean of each technical replicate as a dot in (**A**) and (**D**). Mean ± SEM of each analyzed replicate is shown with a dot representing one analyzed image in (**B**) and (**E**). EAN was induced in three independent experiments (n=4 per group).

**Supplemental Figure 4. ActIIR inhibition does not alter inflammatory cytokine mRNA expression.** Quantitative real-time PCR for pro-inflammatory ((IL-6 (**A**), TNF-α (**B**) and IL-1ß (**C**)) and anti-inflammatory cytokines ((IL-4) (**D**) and IL-10 (**E**)). Sciatic nerves of n=8 rats per group were compared. EAN was induced in two independent experiments with n=4 per group each. Data are plotted as mean ± SEM. An unpaired t-test with Welch’s correction was used.

**Supplemental Figure 5**. **Myelination in EAN recovery is unaffected by ActIIR inhibition.** (**A**) Representative immunostainings for S100+ and N160/200+ axons of sciatic nerves of sham-treated and anti-ActIIR-AB-treated rats at day 30 post immunization (dpi). Scale bar: 50 µm. Co-stained axons were counted as myelinated neurons. The nested analysis of the percentage of myelinated neurons is depicted in (**B**) and (**C**). Overall, n=96 images for sham and n=106 images of the treatment group were quantified. (**D**) shows an exemplary fluoromyelin immunostaining of the sciatic nerve. Scale bar: 50 µm (**E**) and (**F**): Nested analysis of the percentage of the fluoromyelin positive area of the sciatic nerve (sham: n=107, 5 mg/kg anti-ActIIR-AB: n=92 quantified images). Images of n=12 rats per group from distal and proximal nerve stumps were taken randomly at a 20x or 40x magnification. A nested t-test was applied, with data plotted as the overall mean (dashed line), and the mean of each technical replicate as a dot in (**B**) and (**E**). Mean ± SEM of each analyzed replicate is shown with a dot representing one analyzed image in (**C**) and (**F**). EAN was induced in three independent experiments (n=4 per group).

**Supplemental Figure 6. Related to Figure 5. Additional data on muscle fiber preservation induced by ActIIR-AB treatment.** (**A**) Nested analysis of the percentage of fast twitching muscle fibers of the tibialis anterior muscle. Overall, n=116 images for the sham-treated rats and n=111 images of the treatment group were analyzed (n=12 rats per group). (**B**) Minimal feret’s diameter plotted as truncated violin plot showing the median (solid line) and the quartiles (dashed lines). (**C**) Histogram showing the relative frequency distribution of minimal Feret’s diameter fractions, demonstrating a rightward shift indicative of increased muscle fiber size for the treatment group. (**D**) Nested analysis of the percentage of muscle cells with central nuclei and Pax7 expressing muscle fibers (**E**) as markers of muscle hypertrophy. For quantification of (**B**, **C** and **D**, **E**), images of n=12 rats per group were taken randomly at a 20x magnification. Overall, n=14100 muscle fibers for the sham-treated rats and n=8263 muscle fibers of the treatment group were analyzed. An unpaired two-tailed Mann-Whitney test was used for (**B**). A nested t-test was applied for (**A**), (**D**) and (**E**). Mean ± SEM of each analyzed replicate is shown with a dot representing one analyzed image. EAN was induced in three independent experiments (n=4 per group). ****P < 0.0001.

**Supplemental Figure 7. Related to Figure 6. Additional proteomic data.** (**A**) WikiPathways enrichment for whole proteomic data of sham- versus 5 mg/kg anti-ActIIR-AB-treated rats at 30 dpi (days post immunization) using the STRING (version 12.0) platform. (**B**) Volcano plot of differentially expressed proteins in tibialis anterior muscles of anti-ActIIR-AB-treated rats at 30 dpi versus rats at disease peak (18 dpi). Blue colored circles mark significantly upregulated proteins and orange colored circles significantly downregulated proteins at P < 0.05 in the ActIIR-AB-treated rats. (**C**) Volcano plot of differentially expressed proteins in tibialis anterior muscles of sham-treated rats at 30 dpi versus rats at EAN disease peak (18 dpi). Significantly downregulated proteins at P < 0.05 in the sham-treated animals are shown in orange. For proteomic analysis, n=4 rats per group at 30 dpi and n= 3 rats at 18 dpi were used. Student’s t test was used to test for significant (p <0.05) changes in protein abundance.
